# Impacts of Nursing Work Environment on Turnover Intentions: The Mediating Role of Burnout in Ghana

**DOI:** 10.1155/2022/1310508

**Published:** 2022-02-27

**Authors:** Collins Atta Poku, Ernestina Donkor, Florence Naab

**Affiliations:** ^1^Department of Nursing, Kwame Nkrumah University of Science and Technology, Kumasi, Ghana; ^2^School of Nursing and Midwifery, University of Health and Allied Sciences, Ho, Ghana; ^3^School of Nursing and Midwifery, University of Ghana, Accra, Ghana

## Abstract

**Background:**

The nursing practice environment supports excellence and decent work and has the influence to entice and retain the quality nursing workforce. Appreciating the dynamics that affect the turnover intention of RNs offer reasonable solutions to the challenges of the nursing shortage, which directly influence the quality of nursing care. There is a paucity of information on the impacts of these concepts among RNs in Sub-Saharan African. The study therefore aimed at determining the impacts of work environment and burnout on turnover intentions among RNs in Ghana.

**Methods:**

A descriptive cross-sectional design using a simple random and proportionate stratified sampling with a sample of 232 RNs from Municipal and Regional Hospitals, Sunyani, West-Central part of Ghana completed validated instruments measuring work environment, burnout, and turnover intentions. Descriptive analysis was done to find out RNs' perceptions of their work environment and turnover intentions. Mediation analysis by Baron and Kenny's approach was used to determine the mediating effect of burnout on the relationship between the domains of PPE and the turnover intention of RNs. STROBE checklist was used as the reporting tool.

**Results:**

While most RNs had a positive perception about their work environment, greater number of them had turnover intentions. There were significant associations between some nursing work environment facets and turnover intention. The results also showed a statistically significant relationship between nurse-physician relation (*β* = .353, *t* = 5.476, *p* ≤ .001), nurse manager leadership (*β* = −0.485, *t* = −8.192, *p* ≤ .001), nursing foundation for quality care (*β* = .400, *t* = 7.059, *p* ≤ .001), staffing and resource adequacy on (*β* = 0.485, *t* = 8.183, *p* ≤ .001), and turnover intention as mediated by burnout.

**Conclusion:**

Burnout resulting from an unsafe work environment impact RNs' turnover intention. This phenomenon can potentially affect the human resource management and quality of nursing care. Policy strategies aimed at ensuring a professional practice environment and decreased burnout can therefore improve retention of RNs at their workplace.

## 1. Background

Globally, the quality of the nursing workforce and the existence of a professional practice environment (PPE) are in close association with client care satisfaction, quality of care delivery, and positive staff job outcomes [1,2]. The subject of staff turnover is an important area in every health care setting, and it needs thorough research to sustain the evidence-based nursing practice in health care organizations, especially in low-resourced countries.

It is reported that health facilities with a positive work environment and adequate nursing workforce had improved outcomes for both patients and nursing staff [3–5]. The evaluation of quality care and job satisfaction by Registered Nurses (RNs) in the high-resourced countries ranges from worst to best. It is noteworthy to know that countries using the Magnet Certification to encourage value-added practice environments such as the United States have improved in their provision of quality care to patients and other job outcomes [6].

Among the major challenges of low-and-middle-income countries (LMICs) in the work environment is an unbalanced nursing workforce mix with its corresponding negative effects on job outcomes (job dissatisfaction, higher staff turnover, and poor quality of care delivery). Studies have shown a higher incidence of turnover intentions of RNs in LMICs [7,8], though the same cannot be said about the high-resourced countries [9]. This phenomenon, if not effectively tackled, can cause a lot of challenges for health care providers in the long run globally.

Staff turnover is the process by which workers vacate their job or are transferred from the worker's employment [10]. Health labor force scarcities have consequences on global health care delivery and quality of patients' care; appreciating the challenges of turnover rate and retention of staff is essential on the discussion of policy strategies for improving the nursing workforce. Institutions where staff freely express their plans of quitting their job usually have higher turnover [11,12]. High staff turnover intentions in many organizations are attributed to factors such as poor quality of staffing and inadequacy of working material to care for the patient [13–15]. Such poor work conditions present high work demand with low RNs' autonomy over their job, inadequate group support, and increased physical and emotional work demand. Lack of support from nurse managers, unjustified workloads, and increased emotional exhaustion of RNs mostly lead to increased staff intentions of resignation [10–12]. Moreover, most RNs leave their job owing to a lack of professional/career development and poor organizational climate [12,13]. Nursing leadership consciousness of the reasons behind turnover intention can help improve the organizational culture [14,15]. It can also inform nurse managers and administrators about important pillars in health care delivery that makes the nursing workforce satisfied [10,16,17].

In high-resourced settings, though RNs' turnover intentions are low, the lack of leadership support, inadequate involvement of RNs in hospital affairs, and material and human resource constraints at the workplace are observed to be predictors of RNs' turnover intention. Again, nurses' emotional exhaustion and perception of job engagement are significant mediators of nurses' turnover intentions in an organization [18,19].

In LMICs settings such as Ghana, where turnover intention for workers is projected to be 25.9%, increased burnout, limited prospects in the area practice, and bias in career upgrade are cited as the reason for turnover intentions. Burnout among staff who remain to work in the practice environment [20–22] accounts for the high turnover of health staff in the health care settings.

The high turnover intention in any organization presents its challenges, notably is high monetary loss. Moreover, the financial costs of RNs quitting their job in organizations are projected to be higher than before [23–25]. With a very challenging nurse-patient ratio in Ghana, there is still a reportedly high rate of turnover intention and/or turnover among skilled workers [26,27]. With the health care industry depending on the few remaining RNs, the challenge of burnout also emanates.

The problem of high staff turnover rate is an important area in every health care setting, and it needs thorough discussion in the quest to sustain quality in the health care industry. Addressing this problem can fix the quality of patient care challenge in most health facilities. Anecdotally, more RNs are leaving their job either for greener pastures in high-resource countries and/or joining other professions due to the associated challenges in working in public health facilities in Ghana. Contrariwise, there is limited data on the Ghanaian nursing work environment and burnout; and its implications on turnover intentions among RNs. The study, therefore, assessed the influence of work environment and burnout on turnover intentions among RNs in Ghana. Thus, the study sought to ascertain RNs' intention to leave their job, assess the relationship between work environment and turnover intention of RNs, and determine the predictors of turnover intentions among RNs. Findings can inform policies to reduce nursing workforce turnover, thereby addressing unhealthy practice environment, burnout, and quality of patient's care challenges, especially in sub-Saharan Africa.

### 1.1. Theoretical Framework

The Causal Model of Professional Nursing Practice Environment and Nursing Job Outcome was proposed and used by Panunto and Guirardello [28]. The model assumes that the nature of the practice environment impacts the nursing job outcomes when mediated by burnout [29,30]. It seeks to clarify the work environment and ways to improve it. The model is composed of exogenous variables PPE (autonomy, control over work environment, nurse-physician relations) and endogenous variables (job satisfaction, quality of patient care, and intention to leave their job) as mediated by burnout (emotional exhaustion, depersonalization, personal accomplishment). Autonomy, control over the work environment, and nurse-physician relations influence each other while they individually impact the burnout dimensions and nursing job outcomes [28]. Theoretically, the three elements of the PPE were operationalized based on Lake's Practice Environment Scale on Nursing Work Index [31]. Panunto and Guirardello used these designations to replace the features of their model; nurse manager ability, leadership, and support (control over work environment) were described as the capacity to manage resources, support nurses, and the practices needed to deliver quality care. Collegial nurse-physician relationships (i.e., nurse-physician relations) reflected the total worth of the relationship between nurses and physicians. Nurse participation in hospital affairs (i.e., control over work environment) was explained as the degree by which nurses were involved in decision-making regarding healthcare management. Adequate staffing and resources (i.e., control over work environment) measure the availability of human and material resources to meet quality patient care. Nursing foundations for quality care (autonomy) reflected the professionalism of nursing in outlining the standards by which care is given to patients rather than depending on medical models to provide care. The interaction of these features was suggested to impact emotional exhaustion, depersonalization, or personal accomplishment, and in effect, results in the nursing job outcomes; thus, quality nursing care, job satisfaction, and intent to leave a job [32]. The causal model encodes appropriate knowledge about the tendency of specific occurrences to cause other event types. Thus, this model is used to determine the causes of RNs turnover, given assumptions about poor work environment and burnout. The model is, however, complex and cannot handle some situations. The weaknesses also include the issue of multiple roles elements, elements that affect more variables in the model. This is demonstrated in the interactions of the various facets of the practice environment, dimensions of burnout, and nursing job outcomes.

## 2. Methods

### 2.1. Study Design and Setting

A cross-sectional survey was adopted to assess the participant's views on the influence of work environment and burnout on turnover intentions of RNs in Sunyani Municipality, which is found in the middle belt, West-Central part of Ghana, with a population of 123,224 [33]. The population involved 550 RNs in public health facilities who agreed to take part in the research. RNs with at least one year of postqualification experience were included in the study.

### 2.2. Sample Size and Sampling Method

**Figure 1:**
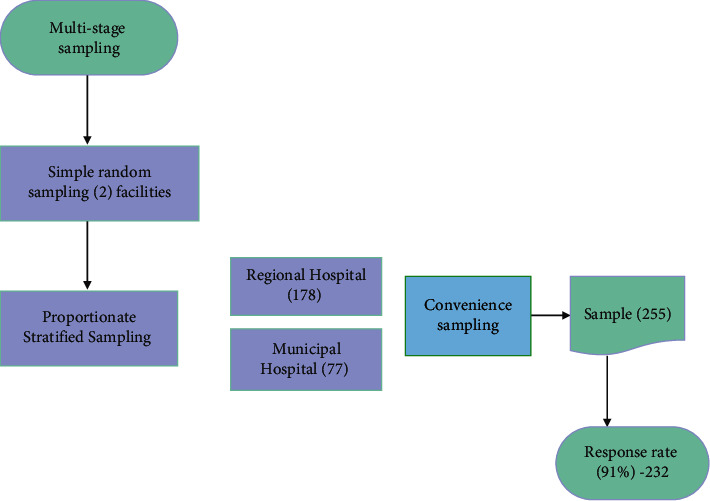
Recruitment flowchart.

A sample of 255 RNs was used as estimated using Yamane's [34] formula for calculating sample size. The recruitment flowchart is presented in Figure 1. Participants were drawn through a multistage sampling method; thus, a simple random sampling technique was used to select two [2] hospitals from seven public facilities in Sunyani for the study. A proportionate stratified sampling approach was then used to assign a proportionate number to each facility based on the nursing workforce of the two settings to ensure unbiased representation (Sunyani Municipal Hospital – 77 and Regional Hospital –178). A convenience sampling was used in recruiting the participants throughout all three shifts run by the RNs in the two hospitals. It was ensured that questionnaires were adequately completed before collection. There was a 91.0% response rate.

### 2.3. Data Collection

The instruments used for the study were standard tools. The instrument comprised of four [4] subsections: sociodemographic information and three [3] other scales. The Practice Environment Scale of Nursing Work Index (PES-NWI) was adapted to measure the nursing work environment. The PES-NWI comprises 32 items divided among five subscales measured on a four Likert scale 1–4 (1 = strongly disagree; 4 = strongly agree). The sum and average of the scales score provide the PES-NWI score. Other studies that have used the PES-NWI have established reliability coefficient between 0.84 and 0.92 [29,35]. Burnout was measured using the Maslach Burnout Inventory (MBI) Scale [36]. MBI consists of 21 items on a Likert scale of 1–7 (1 = Never; 7 = Everyday). Burnout is the sum of all 21 items. Several studies have shown acceptable tool reliability and validity [27,37]. A single-item tool was used to measure the turnover intentions of RN on a Likert scale of 1–4 (1 = strongly disagree; 4 = strongly agree).

Ethical approval was sought from Noguchi Memorial Institute for Medical Research Institutional Review Board–IRB (CPN 045/16–17) and clearance from the Ghana Health Service (GHS) Regional Health Directorate (Brong Ahafo) before the commencement of the research. Consent was obtained from the respondents before the questionnaire was given to them. The benefits and possible risks were also explained to the respondents. Additionally, respondents' anonymity and confidentiality were assured by indicating that they were not required to write their name on the questionnaire and by assuring them that their responses would not in any way be linked to them.

### 2.4. Data Analysis

Statistical Package for Social Sciences (SPSS), version 25.0, was used for the statistical analysis. Data were analyzed at a significance level of 0.05 and a power of 95%. Descriptive analysis was done to find out the frequencies, percentages, mean and standard deviation of RNs' perceptions of practice environment and turnover intentions. The hypothesized model was tested by mediation analysis using [38] Baron and Kenny's approach. The mediation analysis requires the conditions that: practice environment (the predictor) must be significantly associated with turnover intention (the outcome); practice environment (the predictor) must be significantly associated with burnout (the mediator); finally, the turnover intention is regressed on both practice environment (the predictor) and burnout (the mediator). If the effect of the predictor (practice environment) on the outcome (turnover intention) with the presence of the mediator (burnout) is zero, then there is full mediation. If all the conditions are satisfied except for the final condition, then there is a partial mediation. The predictor variable (practice environment), the mediator (burnout), and the outcome variables (turnover intention) were normally distributed and linear relationships existed among them.

## 3. Results

### 3.1. RNs' Perception of Work Environment

The means and standard deviations for the study variable of PPE are found in Table 1. RNs reported a relatively moderate perception of PPE (65.87). Concerning the individual subscales, there were moderate perceptions of nurse manager leadership, ability and support (mean = 21.98), collegial nurse-physician relation (mean = 15.17), staffing, and resource adequacy (mean = 15.47), nurses' participation in hospital affairs (mean = 7.03), and nursing foundation for quality of care (mean = 8.71).

The result shows the mean score for RNs' intention to leave the job to be 2.94 (SD = 1.07), which is high and indicates that RNs had turnover intention, while 39.2% of the nursing workforce had intentions to leave the job, about one-third (31.5%) were indecisive on their intentions to leave the job.

### 3.2. Mediation Effect of Burnout on the Relationship between Work Environment and Turnover Intention

Baron and Kenny (1986) proposed a four-step approach in which several regression analyses are conducted and the significance of the coefficients are examined at each step. In step one, the linear regression analysis was used to establish the relationship between the domains of the work environment (nurse manager leadership, ability and support; nurse-physician relations; nurse participation in hospital affairs; staffing and resource adequacy; nursing foundation for quality care) and turnover intention. The results showed a statistically significant relationship between all the domains of work environment and turnover intention (*p* > .05); except nurse participation in hospital affairs.

Step two sought to establish the relationship between the domains of the work environment (independent variables) and burnout (mediating variable). The results also showed a statistically significant relationship between all the domains of work environment and burnout (*p* > .05). In step three, there was a statistically significant relationship between all the domains of the work environment except one and the turnover intention of the RNs. These findings imply that all the independent variables met the assumptions for a mediation analysis as required by Baron and Kenny (1986), except for nurse participation in hospital affairs.

### 3.3. Mediation Effect of Burnout on the Relationship between Nurse-Physician Relation and Turnover Intention

The results in Table 2 below revealed a complete mediating effect of burnout on the relationship between nurse-physician relationship and turnover intention. The regression coefficients in model one of the mediation analyses initially revealed a significant relationship between the nurse-physician relationship and turnover intention (*β* = −0.130, *t* = −1.993, *p* = 0.048), indicating a significant total effect of the nurse-physician relationship on turnover intention. With the inclusion of the mediating variable (burnout) in model two of the mediation analysis, there was a significant relationship between burnout and turnover intention (*β* = 0.353, *t* = 5.476, *p* ≤ .001). However, the indirect effect of the nurse-physician relationship on turnover intention became insignificant (*β* = −0.026, *t* = −0.399, *p* = .690). This implies that the influence of the nurse-physician relationship on turnover intention is completely mediated by burnout.

### 3.4. Mediation Effect of Burnout on the Relationship between Nurse Manager Leadership Styles and Turnover Intention

**Figure 2:**
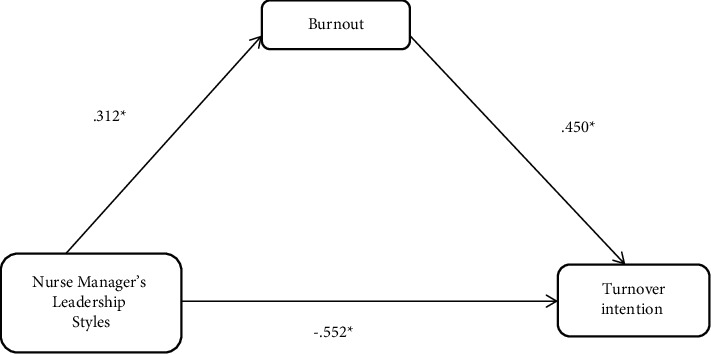
Standardized regression coefficients of the relationship between Nurse Manager's Leadership Styles and turnover intention as mediated by burnout.

In assessing the regression coefficients of the mediation effect of burnout on the relationship between Nurse Manager's Leadership Styles and turnover intention in Figure 2, the first model revealed a significant relationship between Nurse Manager's Leadership Styles and turnover intention (*β* = −0.553, *t* = −.10.038, *p* ≤ .001). When the mediating variable (burnout) was included in the analysis in model two, the indirect effect of Nurse Manager's Leadership Styles on turnover intention remained significant, but with a reduced effect size (*β* = −.485, *t* = −8.192, *p* ≤ .001). This implies that the relationship between Nurse Manager's Leadership Styles on turnover intention is partially mediated by burnout.

### 3.5. Mediation Effect of Burnout on the Relationship between Nursing Foundation for Quality Care and turnover intention

The results of the impact of burnout on the relationship between the Nursing Foundation for Quality Care and turnover intention are presented in Table 3. In assessing the regression coefficients of Nursing Foundation for Quality Care on turnover intention in model one, the results revealed a significant relationship between Nursing Foundation for Quality Care and turnover intention (*β* = .449, *t* = 7.630, *p* ≤ .001). When the mediating variable (burnout) was included in the analysis in model two, the indirect effect of Nursing Foundation for Quality Care on turnover intention through burnout remained significant (*β* = .400, *t* = 7.059, *p* ≤ .001), with a reduced effect size. This implies that burnout partially mediates the relationship between Nursing Foundation for Quality Care and turnover intention.

**Figure 3:**
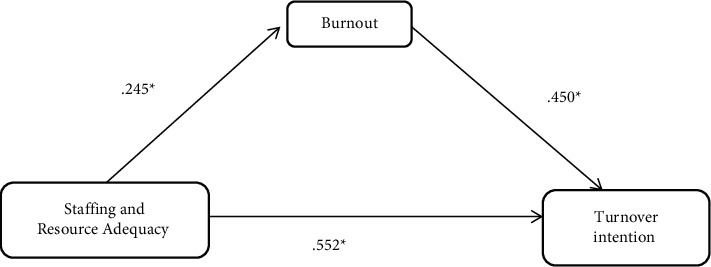
Standardized regression coefficients of the relationship between Staffing and Resource Adequacy and turnover intention as mediated by burnout.

The regression coefficients in model one of the mediation analyses as found in Figure 3 initially revealed a significant relationship between the Staffing and Resource Adequacy and turnover intention (*β* = .552, *t* = 10.031, *p* = .048). This indicates a significant total effect of Staffing and Resource Adequacy on turnover intention. With the inclusion of the mediating variable (burnout) in model two of the mediation analysis, the relationship between burnout and turnover intention was significant (*β* = .164, *t* = 2.774, *p* = .006). The indirect effect of Staffing and Resource Adequacy on turnover intention was still insignificant but with a reduced effect size (*β* = .485, *t* = 8.183, *p* = .006), implying the influence of Staffing and Resource Adequacy on turnover intention.

## 4. Discussion

The turnover intention of the RNs is influenced by the domains of the work environment, and this relationship is mediated by burnout. This finding proves that the work environment has a direct significant relationship with RNs' turnover intentions, and it is consistent with a study that concluded nurse-physician relationship, nursing leadership, staffing, and resource adequacy, and nursing foundation for quality care to be correlated with RN intention to leave [9]. Nurse Managers' leadership styles safeguard quality practice environments and, for that matter, have a greater rate of retaining staff. The findings thus support the assertion that nurse manager ability, leadership, and support impact RNs' intention to stay [29,33]. There is a significant influence of the nurse-physician relations on turnover intention of the RN when burnout mediates the relationship. This implies a likelihood of RNs requesting a transfer or may have high intentions to leave an organization when there is a poor relationship with a physician [39]. This perhaps explains why the increase in poor nurse-physician relations corresponds with the intention of RN to leave a job. The relationship between staffing and resource adequacy and turnover intention nurses is fully mediated by burnout. This result agrees with findings from previous studies [40,41]. Implementing a well-designed, effective staffing plan in the workplace will go a long way to reduce turnover in an organization.

Again, the high turnover of RNs presents many challenges in the care of patients. The study reported RNs generally having positive perceptions about their practice environment. The study agrees with what was reported by [42], indicating encouraging views on most features of their work environment. There were, however, poor perceptions on some facets of the practice environment. With the recent report of inadequate nursing staffing and material resources, it is essential to evaluate the reason behind RNs' intention to leave their profession [37,43,44]. From the current study, RNs had a higher intention of leaving their profession. This position is not different from a study on RNs in South Africa which indicated that about half of all RNs have the intention of leaving their job within two years [45]. Similar findings of a higher rate of turnover intentions have also been observed across the globe [28, 32, 46]. This, however, contradicts finding among RNs in Europe, which projected RNs having lower turnover intention [9]. The differences in the findings can be attributed to the setting of the studies. While the current study was carried out in a low-middle-income country, the previous studies were conducted in countries with more advanced economies. Indeed, practice environment factors in the European hospitals may be different from the sub-Saharan African health settings.

Healthcare delivery in recent times is extremely specialized, and therefore, highly experienced nurses are needed in the healthcare settings. Nursing managers should provide insightful leadership that can be useful in creating and maintaining a work environment that supports the nursing workforce. This may include focusing attention on burnout reduction strategies; thus, improving the availability of both human and material resources in the clinical settings, advocating for a healthy nurse-physician relation, and also improving RNs' participation in hospital affairs [47].

From the above discussion, it suggests that in discussing policy strategies on nursing workforce retention, the overall domains of work environment and burnout impact the turnover intention in many settings at most times. Health care administrators should, therefore, expand the discussion beyond financial incentives as a way of tackling the increased turnover of RNs [48,49]. The resultant effect will be improving nursing job outcomes through the retention of skilled and experienced RNs, which ultimately will help improve the provision of quality nursing care to the patient.

### 4.1. Limitations of the Study

Though the model contained the quality of care and job satisfaction constructs, the questionnaire did not capture the influence of the work environment on both constructs, which is an important aspect to investigate in future research. Moreover, as a constraint for all socially inclined studies [50], the questionnaire was not absolutely and culturally sensitive to determine the true opinions of participants. The tool was, however, made clear to obtain the needed data.

## 5. Conclusion

A poor work environment coupled with burnout increases RNs' turnover intentions. Though most of the RNs had a positive perception of their practice environment, they also had high turnover intentions. Ensuring a healthy workplace for nurses can significantly reduce burnout; and as such, will help keep experienced RNs. With high turnover intention among RNs in Ghana, there are potential effects on the human resource distribution within the clinical setting in particular, and it can compromise the quality of care given to patients.

## Figures and Tables

**Table 1:** Nurses' perception about their professional practice environment.

PES-NWI subscales	Frequency	Percent	Min	Max	Mean	SD
Nurse manager ability, leadership, and support
Dissatisfied	140	60.3
Satisfied	92	39.7	11	44	21.98	3.56
Collegial nurse-physician relations			7	28	15.17	4.19
Poor	137	59.1				
Good	95	40.9				
Staffing and resource adequacy			7	28	15.47	3.78
Inadequate	129	55.6				
Adequate	103	44.4				
Nurses' participation in hospital affair			3	12	7.03	1.68
Dissatisfied	118	50.9				
Satisfied	114	49.1				
Nursing foundation for quality of care			4	16	8.71	2.07
Availability	89	38.4				
Non-availability	143	61.6				
Total PES score			32	128	65.87	9.68
Dissatisfied	100	43.1				
Satisfied	132	56.9				

(Field Data, 2017). PES: Practice Environment Scale, NWI: Nursing Work Index, SD: Standard Deviation. Turnover intention of the RN.

**Table 2:** Mediation effects of burnout on the relationship between Nurse-Physician Relation and Turnover Intention.

Model variables		Unstandardized		Standardized coefficients	t	Sig.
		Coefficients				
		B	Se	Beta		
1	(Constant)	3.350	0.431		7.774	0.000
	Nurse-physician relation	−0.028	0.014	−**130**	−**1.991**	0.048

2	(Constant)	1.072	.581		1.844	0.066
	Nurse-physician relation	−0.006	0.014	−0.026	−0.399	0.690
	Burnout	0.016	0.003	0.353	5.476	0.000

Dependent variable: Turnover Intention; Mediating variable: Burnout; Criterion level = .05.

**Table 3:** Mediation effects of burnout on the relationship between nursing foundation for quality care and turnover intention.

Model variables		Unstandardized coefficients		Standardized coefficients	T	Sig.
		B	Se	Beta		
1	(Constant)	0.578	0.267		2.164	0.032
	Nursing foundation for quality care	0.212	0.028	0.449	7.630	0.000

2	(Constant)	−0.543	0.333		−1.629	0.105
	Nursing foundation for quality care	0.188	0.027	0.400	7.059	0.000
	Burnout	0.013	0.003	0.293	5.178	0.000

Dependent variable: Turnover Intention; Mediating variable: Burnout; Criterion level = .05.

3.6 Mediating Effect of Burnout on the Relationship between Staffing and Resource Adequacy and Turnover Intention.

## Data Availability

All data generated or analyzed during this study are included in this published article.

## Abbreviations

CHPS: Community-based Health Planning and Services
GHS: Ghana Health Service
LMICs: Low-middle-income countries
MBI: Maslach Burnout Inventory
PES-NWI: Practice Environment Scale of Nursing Work Index
PPE: Professional Practice Environment
RNs: Registered Nurses
SDA: Seventh Day Adventist.

## Additional Points

All methods were performed following the STROBE reporting checklist.

## Supplementary Materials

Supplementary Materials STROBE statement: checklist of items that should be included in reports of observational studies.
